# Response of Severe EV71-Infected Patients to Hyperimmune Plasma Treatment: A Pilot Study

**DOI:** 10.3390/pathogens10050625

**Published:** 2021-05-19

**Authors:** Chonnamet Techasaensiri, Artit Wongsa, Thanyawee Puthanakit, Kulkanya Chokephaibulkit, Tawee Chotpitayasunondh, Ubonwon Charoonruangrit, Somjai Sombatnimitsakul, Pilaipan Puthavathana, Hatairat Lerdsamran, Prasert Auewarakul, Boonrat Tassaneetrithep

**Affiliations:** 1 Department of Pediatrics, Faculty of Medicine Ramathibodi Hospital, Mahidol University, Bangkok 10400, Thailand; chonnamet.tec@mahidol.ac.th; 2 Center of Research Excellence in Immunoregulation, Faculty of Medicine Siriraj Hospital, Mahidol University, Bangkok 10700, Thailand; artit.won@mahidol.ac.th; 3 Department of Pediatrics, Faculty of Medicine, Chulalongkorn University, Bangkok 10330, Thailand; thanyawee.p@chula.ac.th; 4 Department of Pediatrics, Faculty of Medicine Siriraj Hospital, Mahidol University, Bangkok 10700, Thailand; kulkanya.cho@mahidol.ac.th; 5 Department of Pediatrics, Queen Sirikit National Institute of Child Health, Bangkok 10400, Thailand; ctawee@health.moph.go.th; 6 National Blood Bank Center, Thai Red Cross, Bangkok 10330, Thailand; ubonwonnbc@gmail.com (U.C.); tul_somjai@hotmail.com (S.S.); 7 Center for Research and Innovation, Faculty of Medical Technology, Mahidol University, Nakon Pathom 73170, Thailand; pilaipan.put@mahidol.ac.th (P.P.); hatairat.ler@mahidol.ac.th (H.L.); 8 Department of Microbiology, Faculty of Medicine Siriraj Hospital, Mahidol University, Bangkok 10700, Thailand; prasert.aue@mahidol.ac.th

**Keywords:** EV71, hand, foot, and mouth disease, hyperimmune plasma treatment, neurological complication

## Abstract

Hand, foot, and mouth disease (HFMD) is highly prevalent in East and Southeast Asia. It particularly affects children under five years of age. The most common causative agents are coxsackieviruses A6 and A16, and enterovirus A71 (EV71). The clinical presentation is usually mild and self-limited, but, in some cases, severe and fatal complications develop. To date, no specific therapy or worldwide vaccine is available. In general, viral infection invokes both antibody and cell-mediated immune responses. Passive immunity transfer can ameliorate the severe symptoms of diseases such as COVID-19, influenza, MERS, and SARS. Hyperimmune plasma (HIP) from healthy donors with high anti-EV71 neutralizing titer were used to transfuse confirmed EV71-infected children with neurological involvement (n = 6). It resulted in recovery within three days, with no neurological sequelae apparent upon examination 14 days later. Following HIP treatment, plasma chemokines were decreased, whereas anti-inflammatory and pro-inflammatory cytokines gradually increased. Interestingly, IL-6 and G-CSF levels in cerebrospinal fluid declined sharply within three days. These findings indicate that HIP has therapeutic potential for HFMD with neurological complications. However, given the small number of patients who have been treated, a larger cohort study should be undertaken. Successful outcomes would stimulate the development of anti-EV71 monoclonal antibody therapy.

## 1. Introduction

Hand, foot, and mouth disease (HFMD) typically occurs in young children. It develops after an infection with coxsackievirus A16 (CV-A16) or an enterovirus (especially enterovirus A71 (EV71)) [1]. While HFMD is found worldwide with low morbidity and mortality rates, it has become endemic in East and Southeast Asia [2]. Almost all patients self-recover from the disease, but neurological complications, such as aseptic meningitis, brainstem encephalitis, neurogenic pulmonary edema, and cardiac dysfunction, are encountered in EV71-infected patients [3,4].

During the last decade, a correlation between severe complications and the elevation of viremia and cytokine storms was observed [5,6,7,8]. Enterovirus infections are also accompanied by increased levels of proinflammatory cytokines, especially in severe cases [9,10]. Neurological involvement is often accompanied by notable rises in peripheral blood (PB) granulocyte colony-stimulating factor (G-CSF) and monocyte chemoattractant protein-1 (MCP-1); however, higher levels of IFN-γ-inducible protein 10 (IP-10), MCP-1, interleukin (IL)-6, IL-8, and G-CSF are found in cerebrospinal fluid (CSF) than in PB. Encephalitis patients display significantly higher IL-6, IL-8, and IL-10 levels in both sources [11,12]. Cytokine and chemokine levels are significantly higher in patients with complications than in uncomplicated cases [13].

The development of cytokine storms could be a consequence of both innate and adaptive immune responses [14]. Several ex vivo and in vitro studies on cytokines and chemokines from multifactorial origins have highlighted monocytes as a key responder of the innate immune system to EV71 infections [15]. Human monocyte-derived macrophage produces high levels of IL-1, IL-6, IL-8, and TNF-α [16]. *γδ* T cells from an HFMD patient with severe complications were highly sensitive to activation, leading to elevated IFN-*γ* and TNF-α production [9]. Interestingly, EV71-infected PBMC produces only pro-inflammatory cytokines, but not type I interferon [17,18]. In addition, epithelial and neural cells are particularly susceptible to EV71 infection, generating various cytokines and chemokines [19,20].

To date, there is no specific treatment for HFMD patients. Several symptomatic treatments have been utilized [21]. For example, intravenous immunoglobulin (IVIG) could reduce the effects of cytokine storms by suppressing inflammatory cytokines IFN-α, IL-6, IL-10, and IL-13, as well as chemokine IL-8; however, the IVIG treatment was unable to completely reduce deaths in patients with pulmonary edema [22]. Influenza A (H1N1)-infected patients receiving hyperimmune IVIG (H-IVIG) showed a lower viral load and IL-6 level than IVIG-treated patients [23]. In EV71-infected mice, the mortality rate was reduced with H-IVIG treatment [24]. Hyperimmune plasma (HIP) treatment has also been reported to be effective in patients with severe acute respiratory syndrome and coronavirus disease 2019 [25,26]. The clinical responses of severe HFMD patients who receive HIP treatment is still unknown.

In the present research, the efficacy of HIP in treating EV71-infected patients with severe complications was evaluated by following their clinical response after receiving plasma with a high titer of anti-EV71 neutralizing antibody. A positive outcome would encourage the adoption of HIP as a specific treatment for EV71-infected patients presenting severe complications.

## 2. Results

### 2.1. Baseline Clinical Profiles of EV71-Infected Patients

The study enrolled children (n = 6) with EV71 infections and neurological involvement. The infections were confirmed by PCR positive status using primers targeting EV71 5’-UTR and the VP1 region in throat and/or rectal swabs, but not sera. The median age of the children was 26 months (range = 16–66 months), and there was an equal number of boys and girls. The most common symptom was an oral ulcer, followed by fever, rash, myoclonus, and tachycardia (Table 1). The mean blood leucocyte count and hemoglobin concentration were within normal ranges, although the patients displayed upper range levels for neutrophils (Table 2). In CSF, the infiltrated RBC, WBC, and neutrophil counts were high, but the glucose and protein levels were within normal ranges; all samples were bacterial culture negative.

### 2.2. Clinical Response to Intravenous HIP Treatment

Patients were intravenously administered ABO phenotype-matched HIP (neutralizing titer 640) at a dosage of 10 mg/kg body weight over a period of up to 1 h. The baseline neurological signs and symptoms (ataxia, tremor, and myoclonic jerk) were resolved after a single dose of the HIP. All patients recovered completely, and were able to be discharged within the following 24–48 h (Table 3). There was no evidence of neurological sequelae, although one patient developed fever and chills 6 h post-HIP treatment. In follow-up visits, every patient was healthy upon examination on day seven and day 14 post-treatment.

**Figure 1:**
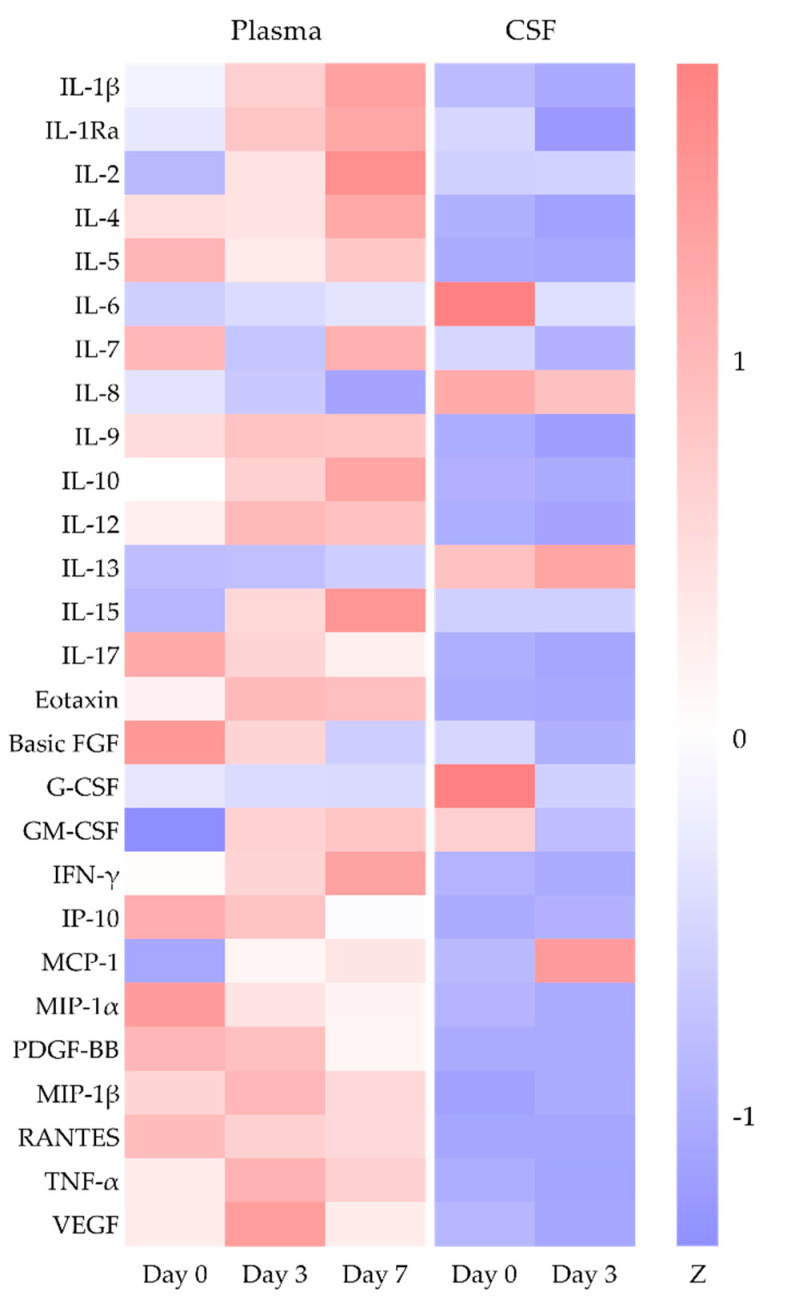
Heat map of cytokine and chemokine levels of recruited EV71-infected patients with neurological manifestation (n = 6). Plasma and CSF were sampled for cytokine, and chemokine assays were conducted prior to treatment (day 0) and on day three and day seven post-HIP treatment. The relative level was normalized to the mean of each cytokine and chemokine. Z, Z-score.

The clinical responses of the patients to intravenous HIP were evaluated by measuring plasma and CSF levels of immune mediators (n = 27) before and on day three and day seven, and before and on day three post-HIP treatment, respectively, employing a multiplex ELISA method (Bio-Rad Laboratories, Inc. Hercules, CA, USA). Following one dose of the HIP treatment, plasma cytokines and chemokines decreased to 1.29%–65.55% by day seven. However, pro-inflammatory cytokines (IL-1β, IFN-γ, IL-6, and TNF-α) and anti-inflammatory cytokines (IL-10 and IL-1Ra) rose slightly (two-fold), while the IL-17 cytokine declined (Figure 1 and Table S1). The levels of all tested chemokines decreased in a time-dependent manner, except for GM-CSF, MCP-1, and Eotaxin. The levels of CSF IL-6, G-CSF, and GM-CSF were suppressed rapidly by day three post-treatment, and significant decreases in the levels of IL1β, IL-1Ra, and IL-7 were observed. However, MCP-1 levels remained high (Figure 1 and Table S2).

## 3. Discussion

Outbreaks of HFMD occur worldwide, with severe complications developing in a number of enterovirus-infected patients, especially children under five years of age. However, the underlying mechanisms remain unknown [27].

Brainstem encephalitis is the most common neurological presentation, accounting for about 60% of neurological manifestations. Some EV71-infected patients with brainstem encephalitis may develop cardiorespiratory dysfunction, which is associated with a high mortality rate (30–40%) during the acute stage of the infection [28]. In the current research, HIP transfusion of EV71-infected children <6 years of age with neurological presentations resulted in their complete recovery without neurological sequalae. All were discharged from the hospital on day three post-treatment. Recovery of severe HFMD patients was reported to range from 1–5 days with IVIG treatment and more than one month without any treatment [29,30,31 ]. In cases presenting with severe neurological manifestations, long-term neurological sequelae can develop [32,33]; these are avoidable if prompt and appropriate treatments are initiated.

The high plasma levels of pro-inflammatory cytokines observed in the present study were previously reported to be present five or more days following the administration of IVIG and immunosuppressive drugs [7]. On the other hand, rapid resolution of cytokine upregulation occurs with IVIG treatment of brainstem encephalitis patients [22]. HFMD patients with brainstem encephalitis or pulmonary edema, or a combination of the two conditions, display diverse plasma levels of IL-8 and TNF-α [34], with IL-8, IP-10, and IL-4 cytokine levels identified as disease progression predictors [10,35]. In the current investigation, the levels of IL-8 and IP-10 decreased after a single HIP transfusion. MCP-1 was previously reported to be lower in severe HFMD cases than in mild HFMD cases [36], consistent with the rise in MCP-1 following HIP treatment observed in the present work. Although the HIP administration resulted in stable plasma IL-4 cytokine production, the pro-inflammatory cytokine levels increased until day seven, and there was an upregulation of IL-10 and IL-1Ra. This implies that there is an immune compensatory mechanism.

The present study reveals that EV71-infected patients had high baseline CSF levels of G-CSF and IL-6, both of which rapidly decreased after the HIP treatment. To the best of our knowledge, there is no previous report on the dynamics of G-CSF and CSF IL-6 levels in IVIG-treated HFMD patients. The upregulation of CSF IL-6, IL-8, and G-CSF correlates with the development of severe complications and, in particular, elevated CSF IL-6 levels, promoting neuronal dysfunction [11,37,38]. In addition, a reduction in viral load was reported in enterovirus-infected mice treated with specific anti-VP1 neutralizing antibody [39]. Thus, HIP treatment might not only reduce the pathology caused by cytokine storms in EV71-infected patients, but also control the viral load via anti-EV71 neutralizing antibodies, as has been observed in patients infected with influenza A virus (H1N1) [40].

## 4. Materials and Methods

### 4.1. Enrollment of Donors and HIP Preparation

Healthy, frequent plasma donors at the National Blood Bank Center, Thai Red Cross Society, Bangkok, Thailand were recruited. Serum was collected for the EV71 sub-genotype B5 (SiICRC10/TH/2011) test, and the determination of the neutralizing antibody titer used a microNT assay [41]. Donors with a high neutralizing titer (≥320) were invited to undergo plasma apheresis. Plasma that was negative for infectious diseases was stored at −40 °C until use.

The research protocol was approved by the Institutional Review Boards of Siriraj Hospital (Si284/2013 and Si726/2013), Ramathibodi Hospital (MURA2013/683), Chulalongkorn Memorial Hospital (69/57), and the Thai Red Cross (NBC 4/2013). Prior written consent was obtained from each plasma donor.

### 4.2. Patients and Study Design

Consent to participate in the study was obtained from the parents/legal guardians of pediatric patients with clinical presentations of severe EV71 infection (Figure S1). All methods were performed in accordance with the approved protocols and guidelines for the diagnosis and treatment of hand, foot, and mouth disease issued by the Department of Medical Services, Ministry of Public Health [42]. Serum and swabs taken from the throat or rectum were collected from each patient to confirm their EV71 infections by routine RT-PCR before the administration of the HIP treatment. Blood and CSF samples were obtained per standard practices. ABO phenotype-matched HIP (neutralizing titer 640) was administered intravenously to each patient at 10 mg/kg over a period of up to 1 h. If necessary, a second HIP dose was given, depending on each patient’s clinical response to the first dose. The clinical responses and adverse events were recorded until discharge.

### 4.3. Measurement of Cytokine and Chemokine Levels

The levels of cytokines and chemokines were measured using a Bio-Plex Pro Human Cytokine 27-Plex Assay Kit (Bio-Rad Lab, Inc., Hercules, CA, USA) in a Bio-Rad BioPlex 200 instrument (Bio-Rad Lab, Inc., Hercules, CA, USA) equipped with Bio-Plex Manager software to determine concentrations (pg/mL) (based on standard curves) of IL-1β, IL-1ra, IL-2, IL-4, IL-5, IL-6, IL-7, IL-9, IL-10, IL-12 (p70), IL-13, IL-15, IL-17, IFN-γ, TNF-α, IL-8, Eotaxin, basic FGF, G-CSF, GM-GSF, IP-10, MCP-1 (MCAP), MIP-1α, MIP-1β, PDGF-BB, RANTES, and VEGF. All measurements were performed in duplicate.

### 4.4. Statistical Analysis

Data were analyzed using GraphPad Prism software version 9.1.0.221 (San Diego, CA, USA). A Kolmogorov–Smirnov test was applied to determine data distribution, and Kruskal–Wallis and Mann–Whitney U tests were used to compare the differences in cytokine and chemokine levels. Probability *p*-values ≤ 0.05 were considered statistically significant in an unpaired *t*-test.

## 5. Conclusions

The presentations of neurological disorders in EV71-infected children (n = 6) were completely controlled within three days by a single (or, at most, two) HIP transfusion(s), with no neurological sequelae observed at the two-week follow-up visit. As the number of patients was small, it was not possible to determine whether the recoveries were due to self-resolution of the disease or the result of the HIP therapy; a larger sample size would be needed to further investigate the effects of HIP. Nevertheless, the findings are promising, and indicate that HIP transfusions may provide a full recovery from the neurological complications of EV71-infected patients. They also suggest that there is a need to develop anti-EV71 monoclonal antibody therapy to prevent unnecessary neurological sequelae in affected children.

## Figures and Tables

**Table 1:** Baseline clinical profiles of patients recruited in the study.

Signs and Symptoms	Number (%) (n = 6)
Oral ulcer	6 (100)
Fever	5 (83)
Myoclonus	4 (67)
Rash	4 (67)
Tachycardia	3 (50)
Nausea/vomiting	2 (33)
Tachypnea	2 (33)
Altered consciousness	1 (17)
Ataxia	1 (17)
Cough	1 (17)
Headache	1 (17)
Hypertension	1 (17)
Running nose	1 (17)

**Table 2:** Laboratory data of the recruited EV71-infected patients with neurological presentations (n = 6).

Laboratory Test	Median (Range)	Normal Range ^1^ Mean (± 2 SD)
CBC	feasurement of Cytokine and Chemokin	
Hemoglobin (g/dL)	11.7 (10.1–12.8)	12.0 (11.5)
WBC count (×10^3^ cell/mm^3^)	11.02 (8.3–15.6)	8.5 (5–15.5)
Neutrophil (%)	55.2 (2.0–73.0)	15–60
CSF		
WBC (cell/mm^3^)	62.8 (8.0–110.0)	0–12 (3)
Neutrophil (%)	35.6 (4–90)	-
RBC (cell/mm^3^)	888 (0–5000)	-
Glucose (mg/dL)	66 (57–78)	-
Protein (mg/dL)	40.5 (6.6–70.0)	79 (23)
CSF bacterial culture	Negative	Negative

^1^ Hospital value.

**Table 3:** Baseline neurological presentations and clinical responses following hyperimmune plasma (HIP) transfusion of the recruited EV71-infected patients.

Patient ID	Baseline Neurological Signs and Symptoms	HIP (Dose)	Clinical Response	Neurological Sequelae
H01PJ	Ataxia	2	Ataxia controlled after first treatment	None
H02SK	Tremor	1	Tremor declined on day one post-treatment	None
H03PP	Ataxia	1	Ataxia controlled	None
H04IS	Myoclonic jerk	1	Myoclonic jerk declined after HIP treatment and was controlled by day three post-treatment	None
H05PU	Ataxia, myoclonic jerk	1	Myoclonic jerk declined after HIP treatment and was controlled by day three post-treatment	None
H06NA	Myoclonic jerk	1	Myoclonic jerk declined after HIP treatment and was controlled by day three post-treatment	None

## Data Availability

All data have been included in the manuscript.

## Supplementary Materials

The following are available online at https://www.mdpi.com/article/10.3390/pathogens10050625/s1, Figure S1: Subject selection and withdrawal, Table S1: Plasma cytokine and chemokine levels of EV71-infected patients with neurological presentations (n = 6) recruited in the study, Table S2: CSF cytokine and chemokine levels of EV71-infected patients with neurological presentations (n = 6) recruited in the study.
