# Supplementary material for: Response of Severe EV71-Infected Patients to Hyperimmune Plasma Treatment: A Pilot Study
Source: Pathogens. 2021 May 19;10(5):625. doi: 10.3390/pathogens10050625 (PMC8161181; doi:10.3390/pathogens10050625)
Supplement: Supplementary file 1 [file pathogens-10-00625-s001.zip › Supplement Figure.pptx]

## Slide 1
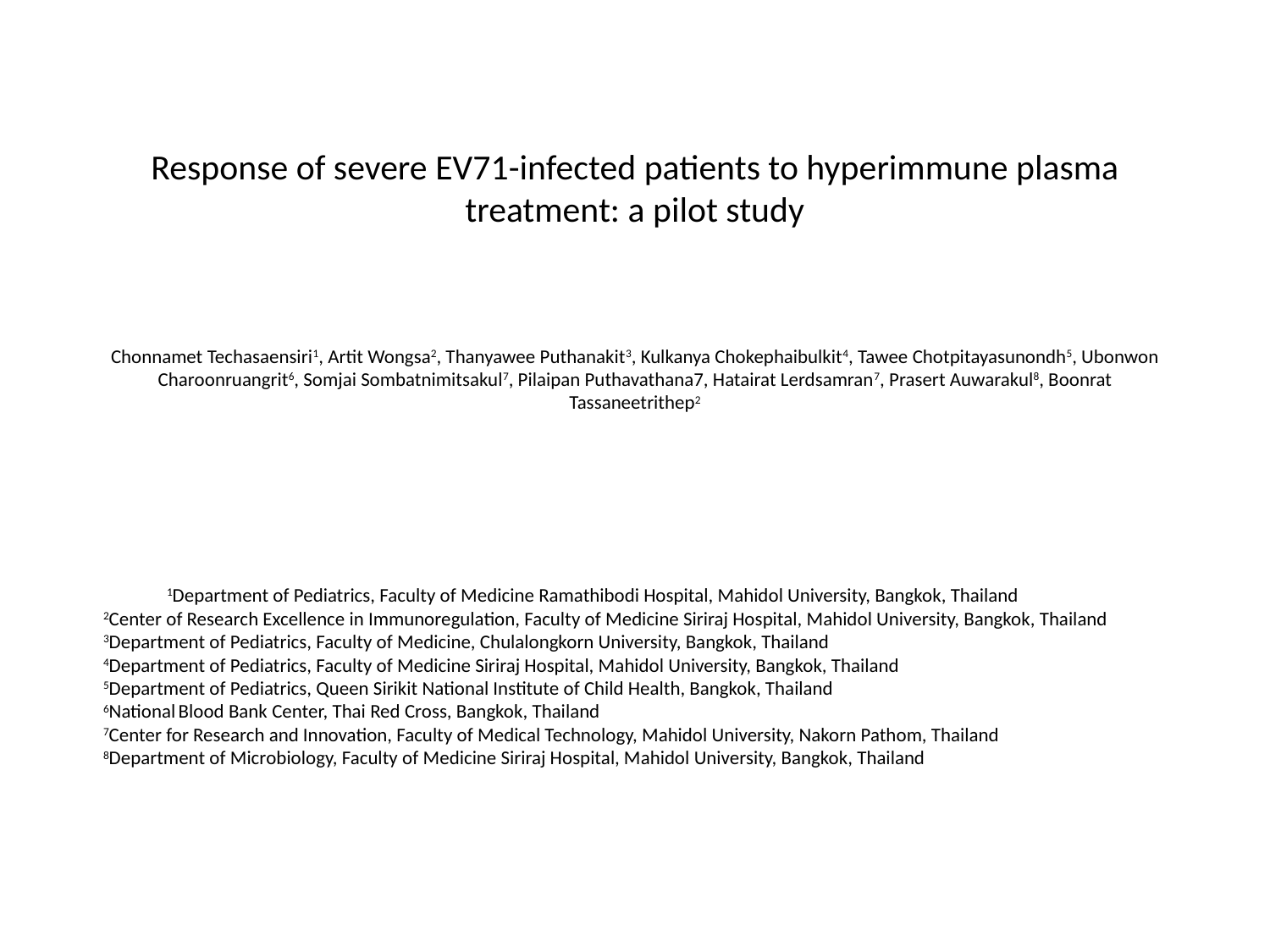

Response of severe EV71-infected patients to hyperimmune plasma treatment: a pilot study
Chonnamet Techasaensiri1, Artit Wongsa2, Thanyawee Puthanakit3, Kulkanya Chokephaibulkit4, Tawee Chotpitayasunondh5, Ubonwon Charoonruangrit6, Somjai Sombatnimitsakul7, Pilaipan Puthavathana7, Hatairat Lerdsamran7, Prasert Auwarakul8, Boonrat Tassaneetrithep2
1Department of Pediatrics, Faculty of Medicine Ramathibodi Hospital, Mahidol University, Bangkok, Thailand
2Center of Research Excellence in Immunoregulation, Faculty of Medicine Siriraj Hospital, Mahidol University, Bangkok, Thailand
3Department of Pediatrics, Faculty of Medicine, Chulalongkorn University, Bangkok, Thailand
4Department of Pediatrics, Faculty of Medicine Siriraj Hospital, Mahidol University, Bangkok, Thailand
5Department of Pediatrics, Queen Sirikit National Institute of Child Health, Bangkok, Thailand
6National Blood Bank Center, Thai Red Cross, Bangkok, Thailand
7Center for Research and Innovation, Faculty of Medical Technology, Mahidol University, Nakorn Pathom, Thailand
8Department of Microbiology, Faculty of Medicine Siriraj Hospital, Mahidol University, Bangkok, Thailand

## Slide 2
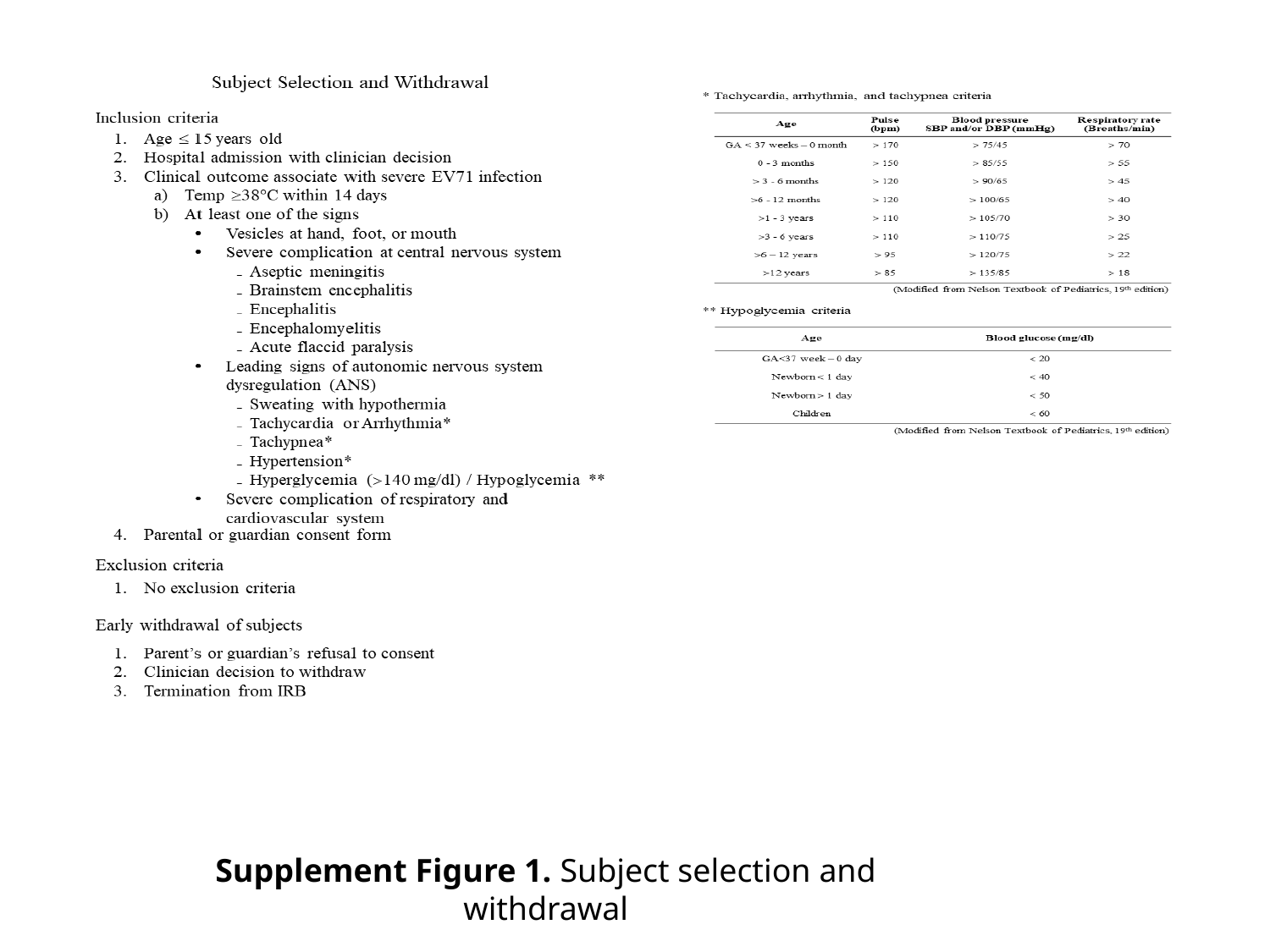

Supplement Figure 1. Subject selection and withdrawal
